# SARS-CoV-2 delta variant infection in domestic dogs and cats, Thailand

**DOI:** 10.1038/s41598-022-12468-y

**Published:** 2022-05-19

**Authors:** Waleemas Jairak, Ekkapat Chamsai, Kitikhun Udom, Kamonpan Charoenkul, Supassama Chaiyawong, Navapon Techakriengkrai, Ratanaporn Tangwangvivat, Kamol Suwannakarn, Alongkorn Amonsin

**Affiliations:** 1 Center of Excellence for Emerging and Re-Emerging Infectious Diseases in Animals, and One Health Cluster, Faculty of Veterinary Science, Chulalongkorn University, Bangkok, 10330 Thailand; 2 Department of Veterinary Public Health, Faculty of Veterinary Science, Chulalongkorn University, Bangkok, Thailand; 3 Bureau of Research and Conservation, Zoological Park Organization, Bangkok, Thailand; 4 Department of Veterinary Microbiology, Faculty of Veterinary Science, Chulalongkorn University, Bangkok, Thailand; 5 Coordinating Unit for One Health, Division of Communicable Diseases, Department of Disease Control, Nonthaburi, Thailand; 6 Department of Microbiology, Faculty of Medicine Siriraj Hospital, Mahidol University, Bangkok, Thailand

**Keywords:** Infectious diseases, Virology

## Abstract

In June–September 2021, we investigated severe acute respiratory syndrome coronavirus-2 (SARS-CoV-2) infections in domestic dogs and cats (n = 225) in Bangkok and the vicinities, Thailand. SARS-CoV-2 was detected in a dog and a cat from COVID-19 positive households. Whole genome sequence analysis identified SARS-CoV-2 delta variant of concern (B.1.617.2). Phylogenetic analysis showed that SARS-CoV-2 isolated from dog and cat were grouped into sublineage AY.30 and AY.85, respectively. Antibodies against SARS-CoV-2 could be detected in both dog (day 9) and cat (day 14) after viral RNA detection. This study raises awareness on spill-over of variant of concern in domestic animals due to human-animal interface. Thus, surveillance of SARS-CoV-2 in domestic pets should be routinely conducted.

## Introduction

Severe acute respiratory syndrome coronavirus-2 (SARS-CoV-2) has been reported to infect several animal species. In Thailand, the first cases of SARS-CoV-2 infection in dogs and cats were reported in May-2021 and have raised a public health concern^1^. To date, SARS-CoV-2 spillover from humans to animals have been reported globally in 32 countries in at least 17 animal species such as cats, dogs, mink, otter, pet ferrets, lions, tigers, pumas, snow leopards, gorillas, white-tailed deer, fishing cat, binturong, coati, hyena, lynx, hippopotamus and hamster (as of January 2022)^2^. In domestic dogs and cats, SARS-CoV-2 infection has been reported in several countries in America, Europe, and Asia^1–9^.

At least five SARS-CoV-2 variants of concerns (VOC) have been classified by WHO including Alpha variant (B.1.1.7), Beta variant (B.1.351), Gamma variant (P.1), Delta variant (B.1.617.2), and Omicron (B.1.1.529)^10^. To date, due to the accumulate mutations of SARS-CoV-2 genome especially the mutations of the spike (S) protein which related to viral entry and binding to host cell receptor have resulted in several SARS-CoV-2 VOCs^11,12^. In Thailand, the delta variant (B.1.617.2) has become a predominant variant during the 4th wave of COVID-19 outbreak since July 2021^13^. As of January 2022, at least 2.3 million confirmed cases with 21,959 deaths have been reported in Thailand^14^.

Due to human-animal interface and close contact with the owners, domestic dogs and cats have high risk of SARS-CoV-2 exposure. Spill-over of the SARS-CoV-2 variant of concerns poses higher risk to domestic animals. The SARS-CoV-2, alpha variant (B.1.1.7) transmission from human to dogs and cats have been reported worldwide during early COVID-19 outbreaks^1,15,16^. Moreover, the SARS-CoV-2, delta variant (B.1.617.2) infection in dogs emerged in the US, Spain and China, and will became more frequent scenario^17–19^. During June–September 2021, the center of excellence for emerging and re-emerging infectious diseases in animals (CUEIDAs), Chulalongkorn University conducted a cross-sectional survey for SARS-CoV-2 in domestic dogs and cats in Bangkok and the vicinities. Nasal, oral, and rectal swabs were collected from domestic dogs and cats for SARS-CoV-2 detection. We identified SARS-CoV-2 RNA from a dog and a cat from COVID-19 positive households. This study is the first to report SARS-CoV-2, B.1.617.2 (Delta variant) infection in dog and cat in Thailand.

## Results

### SARS-CoV-2 infection in dogs and cats

A cross-sectional survey for SARS-CoV-2 in dogs and cats in Bangkok and the vicinities was conducted during June to September 2021. We collected nasal, oral, and rectal swabs from 225 animals (105 dogs and 120 cats) from 199 households. Of 225 animals, 19 animals were sampled from twelve COVID-19 positive households (Table 1). SARS-CoV-2 detection by realtime RT-PCR assay with specific primers and probes for the N2, E and RdRp genes was performed following CDC and WHO recommendations^20,21^. In this study, SARS-CoV-2 RNA could be detected from a cat (n = 1) and a dog (n = 1) from COVID-19 positive households. The positive swab samples were from a cat (CU27516) in Nonthaburi province in July 2021 and a dog (CU27791) in Bangkok in September 2021 (Table 2).

**Table 1:** SARS-CoV-2 survey in COVID-19 households and unknown status households during COVID-19 outbreak in Thailand from June 2021 to September 2021.

Month	COVID-19 Households		Unknown status Households	
	Dog	Cat	Dog	Cat
	#positive/#tested	#positive/#tested	#positive/#tested	#positive/#tested
Jun 2021	0/1	0/8	0/54	0/41
Jul 2021	0/2	1*/5	0/2	0/5
Aug 2021	0/1	0/1	0/25	0/37
Sep 2021	1**/1	0/0	0/19	0/23
Total	1/5	1/14	0/100	0/106

*SARS-CoV-2 positive; July 15, 2021.

**SARS-CoV-2 positive; September, 12, 2021.

**Table 2:** List of COVID-19 positive households where dogs and cats were sampled and tested for SARS-CoV-2.

Household	Sample collection	Province	# of COVID-19 patients in household	Days sample collection from owner 1st positive	#SARS-CoV-2 positive swabs/# swab samples			#SARS-CoV-2 positive animals /# animals	
					Nasal swab	Oral swab	Rectal swab	Dog	Cat
1	Jun 2021	Bangkok	N/A	6 days	0/4	0/4	0/4	0/0	0/4
2	Jun 2021	Samut Prakan	2/2	2 days	0/3	0/3	0/3	0/0	0/3
3	Jun 2021	Bangkok	N/A	4 days	0/1	0/1	0/1	0/0	0/1
4	Jun 2021	Bangkok	N/A	15 days	0/1	0/1	0/1	0/1	0/0
5	Jul 2021	Bangkok	1/1	7 days	0/1	0/1	0/1	0/1	0/0
6	Jul 2021	Bangkok	1/1	15 days	0/1	0/1	0/1	0/0	0/1
7 (A)	Jul 2021	Nonthaburi	3/3	4 days	1/1*	1/1*	0/1	0/0	1/1*
8	Jul 2021	Bangkok	2/2	15 days	0/2	0/2	0/2	0/0	0/2
9	Jul 2021	Pathum Thani	5/5	10 days	0/1	0/1	0/1	0/1	0/0
10	Jul 2021	Bangkok	1/1	2 days	0/1	0/1	0/1	0/0	0/1
11	Aug 2021	Bangkok	2/2	3 days	0/2	0/2	0/2	0/1	0/1
12 (B)	Sep 2021	Bangkok	4/4	5 days	1/1**	1/1**	1/1**	1/1**	0/0
		Total			2/19	2/19	1/19	1/5	1/14

*SARS-CoV-2 positive from household A; July 15, 2021.

**SARS-CoV-2 positive from household B; September, 12, 2021.

**Figure 1:**
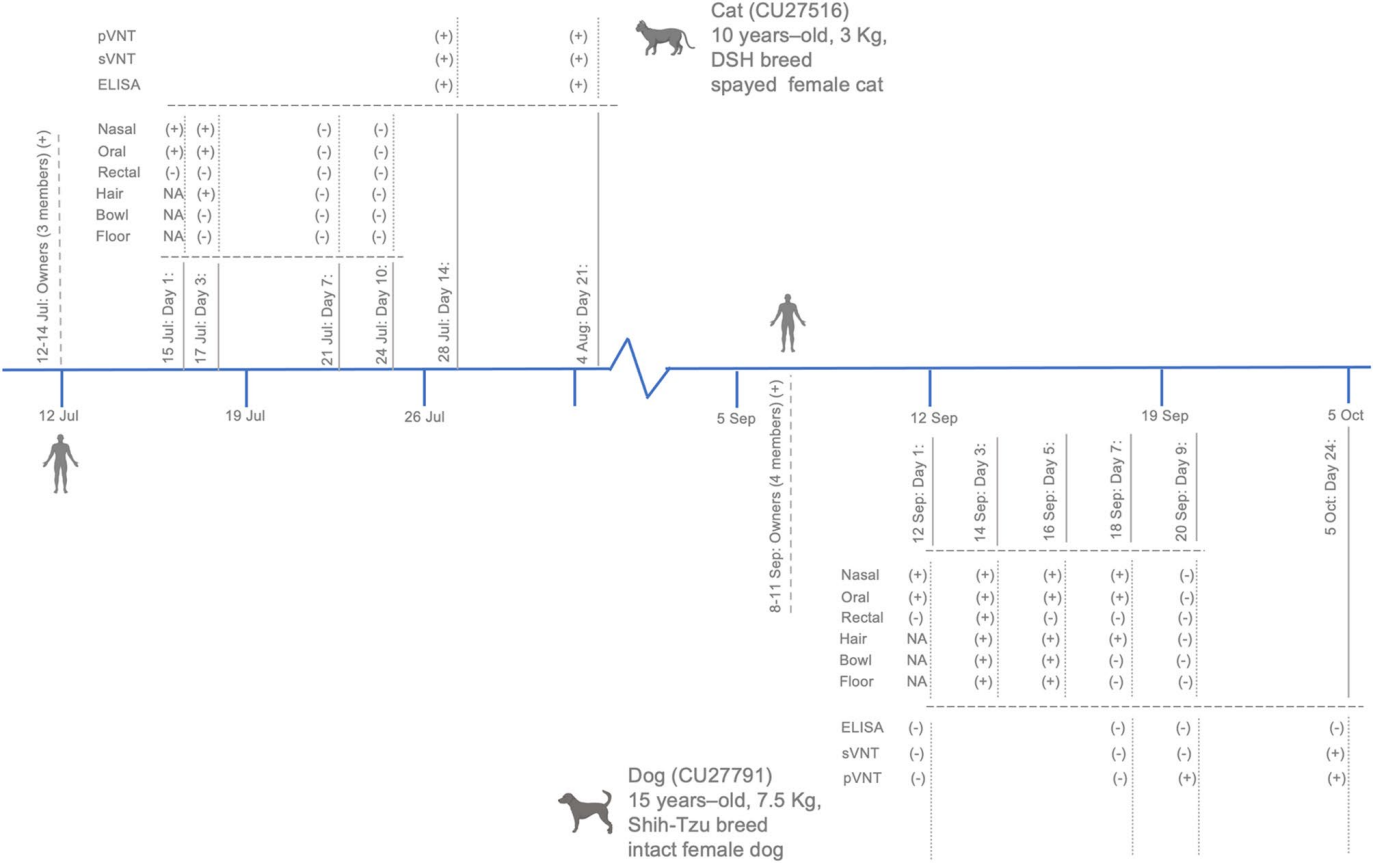
Timeline of SARS-CoV-2 detection in domestic dog and cat from covid-19 positive households in this study.

COVID-19 positive dog and cat were followed up for virological and serological investigation. A cat (CU27516) is 10 years-old, 3 kg, domestic short hair, spayed female cat. A cat was healthy and transferred to private animal hospital on 15 July 2021 (day 1) due to family members (n = 3) were COVID-19 positive and quarantined at the field hospital. All members were tested positive on 12–14 July 2021 and tested negative after 14 days. Swab (nasal, oral and rectal) and environmental samples were collected on four occasions (day 1, 3, 7, and 10). During quarantine, a cat did not show any clinical signs. Nasal and oral swabs tested positive for SARS-CoV-2 RNA at day 1 and day 3 (Ct 20.66–34.36). Low viral RNA (high Ct Value) was detected in animal hair swabs on day 3 (Ct 36.56). All samples tested negative on day 7 and day 10 (Table 3 and Fig. 1). A dog (CU27791) is 15 years-old, 7.5 kg, Shih-Tzu breed, intact female dog. The animal was transferred to private animal hospital on 12 September 2021 due to family members (n = 4) tested positive for COVID-19 on 8–11 September 2021. Sample collection was conducted on five occasions (day 1, 3, 5, 7, and 9). SARS-CoV-2-RNA was detected in nasal and oral swabs of dogs on day 1–7 (Ct 19.06–39.87). The highest viral titers (low Ct values) were observed in nasal and oral swabs on day 3. Viral RNAs were also detected in environmental samples (hair, water container and floor) on day 3, 5, and 7 (Ct 29.87–38.13). A dog did not have fever but showed mild respiratory signs with serous nasal discharge on day 5–7 (Table 3 and Fig. 1).

**Table 3:** Result of SARS-CoV-2 detection from cat and dog swab samples by realtime-RT-PCR.

Date	Realtime RT PCR positive (Ct value)																			
	Nasal			Oral			Rectal			Hair/body swab				Water container swab			Floor swab			
	N^a^	E^b^	RdRp^b^	N^a^	E^b^	RdRp^b^	N^a^	E^b^	RdRp^b^	N^a^	E^b^	RdRp^b^		N^a^	E^b^	RdRp^b^	N^a^		E^b^	RdRp^b^
**Cat CU 27516**																				
15 Jul 21	+ (20.66)	+ (23.66)	+ (28.49)	+ (28.69)	+ (27.28)	+ (31.59)	–	–	–	N/A	N/A	N/A		N/A	N/A	N/A	N/A		N/A	N/A
17 Jul 21	+ (28.70)	+ (28.61)	+ (31.93)	+ (31.90)	+ (30.99)	+ (34.36)	–	–	–	–	–	+ (36.56)		–	–	–	–		–	–
21 Jul 21	–	–	–	–	–	–	–	–	–	–	–	–		–	–	–	–		–	–
24 Jul 21	–	–	–	–	–	–	–	–	–	–	–	–		–	–	–	–		–	–
**Dog CU27791**																				
12 Sep 21	–	+ (31.98)	+ (31.62)	+ (38.50)	+ (33.84)	+ (33.15)	–	–	–	N/A	N/A	N/A		N/A	N/A	N/A	N/A		N/A	N/A
14 Sep 21	+ (19.06)	+ (19.86)	+ (20.43)	+ (27.15)	+ (26.05)	+ (25.48)	+ (38.59)	+ 35.82)	+ (34.71)	+ (34.61)	+ (30.37)	+ (29.87)		+ (35.99)	+ (32.07)	+ (31.33)	–		+ (32.92)	+ (31.68)
16 Sep 21	+ (27.29)	+ (24.45)	+ (23.17)	+ (39.87)	+ (34.82)	+ (31.60)	–	–	–	–	+ (34.36)	+ (31.80)		–	+ (38.13)	–	–		+ (33.33)	+ (31.75)
18 Sep 21	+ (35.75)	+ (31.82)	+ (29.77)	–	+ (37.38)	–	–	–	–	–	+ (35.81)	+ (36.31)		–	–	–	–		–	–
20 Sep 21	–	–	–	–	–	–	–	–	–	–	–	–		–	–	–	–		–	–

^a^Primers and probes specific to N gene ^21^.

^b^Primers and probes specific to E and RdRp genes of SARS-CoV-2 ^20^.

In this study, we collected blood sample from SARS-CoV-2 positive animals (n = 6 serum; cat (CU27516) (n = 2 time points) and dog (CU27791) (n = 4 time points)). We tested serum samples for SARS-CoV-2 antibodies by indirect multispecies ELISA, surrogate virus neutralization test (sVNT) and pseudotyped virus neutralization (pVNT). Our result showed that a cat was positive for SARS-CoV-2 antibodies by indirect ELISA (113%-271%), sVNT (90%-95%) and pVNT (1:640) at day 14 and day 21. While a dog was positive for SARS-CoV-2 antibodies by sVNT (48.81%) at day 24 and pVNT (1:20) at day 9 and day 24 (Table 4).

**Table 4:** Serological test result of protein-based ELISA, sVNT and pVNT for SARS-CoV-2 antibodies in COVID-19 positive cat and dog.

ID	Host	Date	Days sample collection from animal 1^st^ positive	Serological assay				
				ELISA *		sVNT**		pVNT ***
				OD	%SP	OD	% inhibition	Titer
Cat CU27516	Cat							
		28-Jul-21	14 days	1.375	113.64% ( +)	0.199	90.64% ( +)	640 ( +)
		4-Aug-21	21 days	3.219	271.31% ( +)	0.102	95.32% ( +)	640 ( +)
Dog CU27791	Dog							
		12-Sep-21	1 day	0.056	0.63%	2.403	2.85%	< 10 (-)
		18-Sep-21	7 days	0.060	0.96%	2.309	6.65%	< 10 (-)
		20-Sep-21	9 days	0.157	9.06%	2.008	18.82%	20 ( +)
		5-Oct-21	24 days	0.069	1.06%	1.294	48.81% ( +)	20 ( +)

*ELISA: ID Screen® SARS-CoV-2 Double Antigen Multi-species ELISA kit (ID VET, Montpellier, France). For the cutoff values, S/P% ≥ 60% is postive, S/P% 50–60% is suspected, and < 50% is negative.

**sVNT (Virus neutralization test): cPass SARS-CoV-2 Neutralization Antibody Detection Kit (GenScript Biotech, Jiangsu, China). The cutoff values were defined as follows; positive if % inhibition ≥ 20% is positive and negative otherwise.

***pVNT (Pseudotype Virus neutralization test): pVNT *was determined against Lentiviral pseudovirus expressing spike of SARS-CoV-2 strain Wuhan-Hu-1 (Genbank NC_045512).* Serum dilution that gave a 50% reduction in GFP signal (IC50) was considered positive. The titer of ≥ 10 is considered postive and negative otherwise.

### Delta variant of SARS-CoV-2 from dog and cat in Thailand

After SARS-CoV-2 detection, we performed whole genome sequencing from nasal swab of cat (CU27516; Ct 20.66) and dog (CU27791; Ct 19.06) by using the ARTIC multiplex PCR protocol and MinION sequencing platform (Oxford Nanopore Technologies). A total of 173,580 (CU27516) and 223,045 (CU27791) reads were archived and 85.70% (1,309 coverages) and 66.56% (2,296 coverages) were mapped to reference genome, respectively. Whole genome sequences of the viruses were 29,704 (CU27791) and 29,861 (CU27516) nucleotides with 2,296 and 1,309 coverages, respectively. Whole genome sequences of the viruses were deposited in the GenBank (OK555092 and OK539641) and GISAID (EPI_ISL_5320246 and EPI_ISL_5315539) (Supplement Table 1).

**Figure 2:**
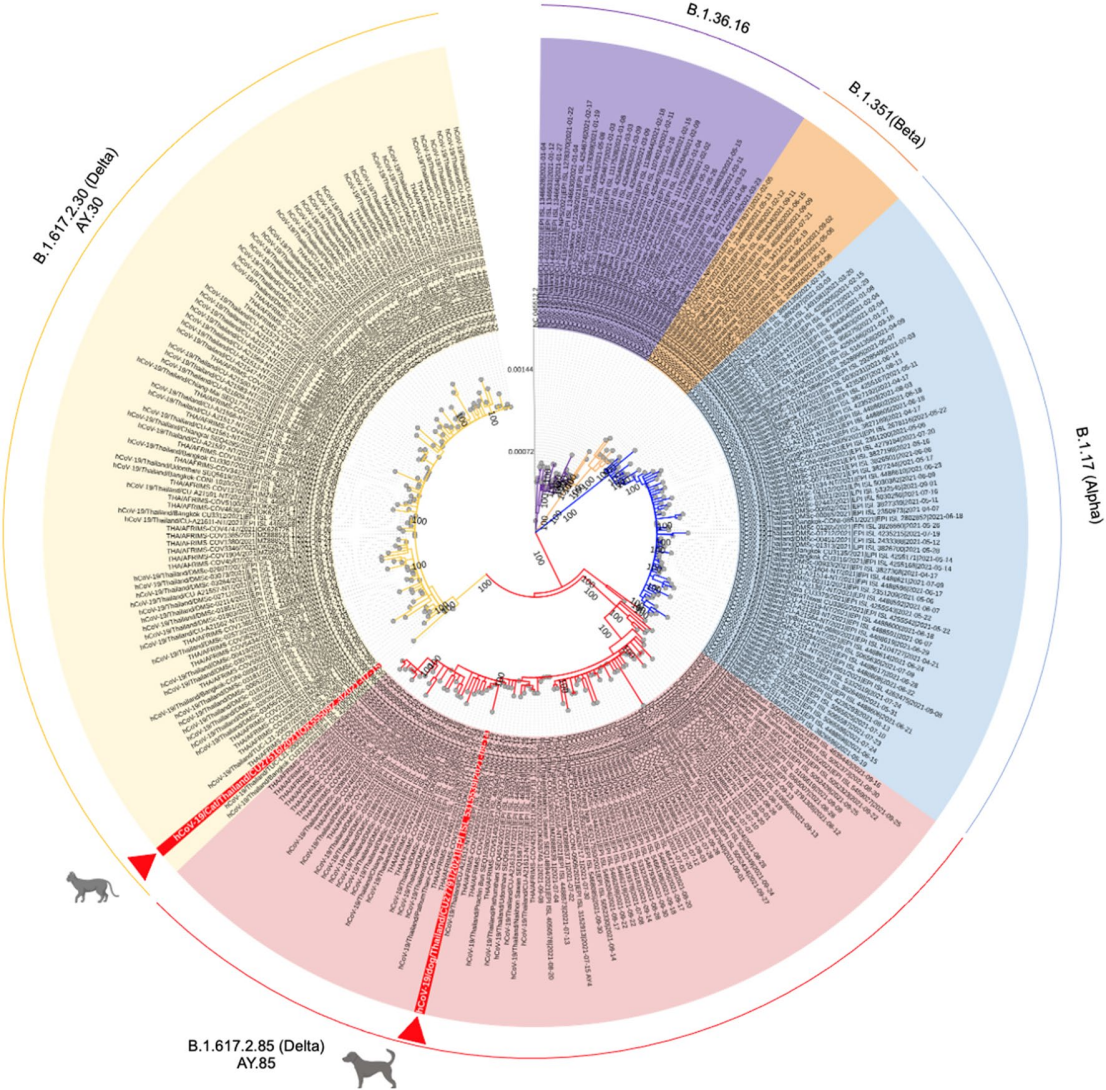
The maximum likelihood tree of SARS-CoV-2 from dog, cat and human from Thailand. Tree was constructed by using IQ-TREE version 2.1.3 (http://www.iqtree.org/) using the GTR + Γ model of nucleotide substitution, default heuristic search options, and ultrafast bootstrapping with 1000 replicates. Tree was visualized by iTOL version 6.0 (https://itol.embl.de/). Colors indicate PANGO lineages including purple (B.1.36.16), orange (B.1.351; Beta), blue (B.1.1.7; Alpha), pink (B.1.617.2.85; Delta AY.85) and yellow (B.1.617.2.30; Delta AY.30). Red arrows indicate SARS-CoV-2 from dog and cat in this study.

Phylogenetic analysis of SARS-CoV-2 was performed by comparing complete genome of SARS-CoV-2 from dog and cat in this study and 289 SARS-CoV-2 genomes available in the GISAID and GenBank database. The sequences were aligned by using the MAFFT FFT-NS-2 algorithm and phylogenetic tree was constructed by using IQ-TREE 2 applying the GTR + Γ model of nucleotide substitution with default heuristic search options and bootstrapping with 1000 replicates. Lineage classification was performed by using the Pangolin tool. Phylogenetic analysis showed that SARS-CoV-2 of dog and cat in this study clustered with human SARS-CoV-2 of B.1.617.2 (Delta variant of concern). The cat and dog SARS-CoV-2 were grouped into sublineage AY.30 (B.1.617.2.30) and AY.85 (B.1.617.2.85), respectively (Fig. 2). BLAST analysis of SARS-CoV-2 whole genome sequences showed that cat and dog SARS-CoV-2 possessed high nucleotide similarities to human SARS-CoV-2 of delta variant in Aug-2021 (99.98%; COV2513/21) and Oct-2021 (99.98%; COV3783/21), respectively (Supplement Table 2). Characteristic mutation analysis on spike protein of SARS-CoV-2 of dog and cat showed identical mutations to those of delta variant (Table 5). The mutations at the N-terminal-domain (NTD) (T19R, E156G, F157del, R158del), the receptor binding motif (RBM) (L452R, T478K), the subdomain 2 (D614G), S1 unit (P681R) and heptad repeat 1 (D950N) were observed. It should be noted that delta variant, AY.85 sublineage contained additional mutation at T95I on N terminal domain (NTD) and it may associate with higher viral load which promote viral transmission from human to human/animal due to close contact.

**Table 5:** Characteristic mutations of SARS-CoV-2 delta variant from cat (AY.30) and dog (AY.85) and reference viruses.

Characteristic mutation (AY.30)															
Virus	GISAID#	Location	Species	Date	Lineage	Amino acid substitution/ deletion ^a^									
						Spike gene									
						T19R	E156G	F157del	R158del	L452R	T478K	D614G	P681R	D950N	
Wuhan-Hu-1	NC_045512.2	China	Human	Dec-19	B	T	E	F	R	L	T	D	P	D	
AFRIMS-COV1370-2021	MZ888533	Thailand	Human	Jun-21	B.1.617.2.30	R	G	157del	158del	R	K	G	R	N	
AFRIMS-COV2513-2021	MZ888556	Thailand	Human	Aug-21	B.1.617.2.30	R	G	157del	158del	R	K	G	R	N	
CUA21611	EPI_ISL_4488568	Thailand	Human	Jul-21	B.1.617.2.30	R	G	157del	158del	R	K	G	R	N	
CU27516^a^	EPI_ISL_5320246	Thailand	Cat	Jul-21	B.1.617.2.30	R	G	157del	158del	R	K	G	R	N	
AFRIMS-COV3783-2021	OK626714	Thailand	Human	Jul-21	B.1.617.2.85	R	G	157del	158del	R	K	G	R	N	
CU27791^b^	EPI_ISL_5315539	Thailand	Dog	Sep-21	B.1.617.2.85	R	G	157del	158del	R	K	G	R	N	

						ORF1a					N				
						P309L	P1640L	H2092Y	V3718A		D63G	L139F	R203M	D377Y	R385K
Wuhan-Hu-1	NC_045512.2	China	Human	Dec-19	B	P	P	H	V		D	L	R	D	R
AFRIMS-COV1370-2021	MZ888533	Thailand	Human	Jun-21	B.1.617.2.30	L	L	Y	A		G	F	M	Y	K
AFRIMS-COV2513-2021	MZ888556	Thailand	Human	Aug-21	B.1.617.2.30	L	L	Y	A		G	F	M	Y	K
CUA21611	EPI_ISL_4488568	Thailand	Human	Jul-21	B.1.617.2.30	L	L	Y	A		G	F	M	Y	K
CU27516^a^	EPI_ISL_5320246	Thailand	Cat	Jul-21	B.1.617.2.30	L	L	Y	A		G	F	M	Y	K
AFRIMS-COV3783-2021	OK626714	Thailand	Human	Jul-21	B.1.617.2.85	P	P	H	V		G	L	M	Y	R
CU27791^b^	EPI_ISL_5315539	Thailand	Dog	Sep-21	B.1.617.2.85	P	P	H	V		G	L	M	Y	R

						ORF3a	M	ORF7a			ORF8				
						S26L	I82T	V82A	L116F	T120I	S84L	D119del	F120del		
Wuhan-Hu-1	NC_045512.2	China	Human	Dec-19	B	S	I	V	L	T	L	D	F		
AFRIMS-COV1370-2021	MZ888533	Thailand	Human	Jun-21	B.1.617.2.30	L	T	A	F	I	L	119del	120del		
AFRIMS-COV2513-2021	MZ888556	Thailand	Human	Aug-21	B.1.617.2.30	L	T	A	F	I	L	119del	120del		
CUA21611	EPI_ISL_4488568	Thailand	Human	Jul-21	B.1.617.2.30	L	T	A	F	I	L	119del	120del		
CU27516^a^	EPI_ISL_5320246	Thailand	Cat	Jul-21	B.1.617.2.30	L	T	A	F	I	L	119del	120del		
AFRIMS-COV3783-2021	OK626714	Thailand	Human	Jul-21	B.1.617.2.85	L	T	A	L	I	L	119del	120del		
CU27791^b^	EPI_ISL_5315539	Thailand	Dog	Sep-21	B.1.617.2.85	L	T	A	L	I	L	119del	120del		

Characteristic mutation (AY.85)															
Virus	GISAID#	Location	Species	Date	Lineage	Amino acid substitution/ deletion ^a^									
						Spike gene									
						T19R	T95I	E156G	F157del	R158del	L452R	T478K	D614G	P681R	D950N
Wuhan-Hu-1	NC_045512.2	China	Human	Dec-19	B	T	T	E	F	R	L	T	D	P	D
AFRIMS-COV1370-2021	MZ888533	Thailand	Human	Jun-21	B.1.617.2.30	T	T	G	157del	158del	R	K	G	R	N
AFRIMS-COV2513-2021	MZ888556	Thailand	Human	Aug-21	B.1.617.2.30	R	T	G	157del	158del	R	K	G	R	N
CUA21611	EPI_ISL_4488568	Thailand	Human	Jul-21	B.1.617.2.30	R	T	G	157del	158del	R	K	G	R	N
CU27516^a^	EPI_ISL_5320246	Thailand	Cat	Jul-21	B.1.617.2.30	R	T	G	157del	158del	R	K	G	R	N
AFRIMS-COV3783-2021	OK626714	Thailand	Human	Jul-21	B.1.617.2.85	R	I	G	157del	158del	R	K	G	R	N
CU27791^b^	EPI_ISL_5315539	Thailand	Dog	Sep-21	B.1.617.2.85	R	I	G	157del	158del	R	K	G	R	N

						ORF1a						N			
						E148G	A1306S	P2046L	P2287S	V2930L	T3255I	D63G	R203M	G215C	D377Y
Wuhan-Hu-1	NC_045512.2	China	Human	Dec-19	B	E	A	P	P	V	T	D	R	G	D
AFRIMS-COV1370-2021	MZ888533	Thailand	Human	Jun-21	B.1.617.2.30	E	A	P	P	V	T	G	M	G	Y
AFRIMS-COV2513-2021	MZ888556	Thailand	Human	Aug-21	B.1.617.2.30	E	A	P	P	V	T	G	M	G	Y
CUA21611	EPI_ISL_4488568	Thailand	Human	Jul-21	B.1.617.2.30	E	A	P	P	V	T	G	M	G	Y
CU27516^a^	EPI_ISL_5320246	Thailand	Cat	Jul-21	B.1.617.2.30	E	A	P	P	V	T	G	M	G	Y
AFRIMS-COV3783-2021	OK626714	Thailand	Human	Jul-21	B.1.617.2.85	G	S	L	S	L	I	G	M	C	Y
CU27791^b^	EPI_ISL_5315539	Thailand	Dog	Sep-21	B.1.617.2.85	E	S	L	S	L	I	G	M	C	Y

						ORF3a		E	M	ORF7a		ORF7b	ORF8		
						S26L	L140F	V62F	I82T	V82A	T120I	T40I	S84L	D119del	F120del
Wuhan-Hu-1	NC_045512.2	China	Human	Dec-19	B	S	L	V	I	V	T	T	L	D	F
AFRIMS-COV1370-2021	MZ888533	Thailand	Human	Jun-21	B.1.617.2.30	L	L	V	T	A	I	T	L	119del	120del
AFRIMS-COV2513-2021	MZ888556	Thailand	Human	Aug-21	B.1.617.2.30	L	L	V	T	A	I	T	L	119del	120del
CUA21611	EPI_ISL_4488568	Thailand	Human	Jul-21	B.1.617.2.30	L	L	V	T	A	I	T	L	119del	120del
CU27516^a^	EPI_ISL_5320246	Thailand	Cat	Jul-21	B.1.617.2.30	L	L	V	T	A	I	T	L	119del	120del
AFRIMS-COV3783-2021	OK626714	Thailand	Human	Jul-21	B.1.617.2.85	L	F	F	T	A	I	I	L	119del	120del
CU27791^b^	EPI_ISL_5315539	Thailand	Dog	Sep-21	B.1.617.2.85	L	F	F	T	A	I	I	L	119del	120del

^a^SARS-CoV-2 characterized in this study.

^b^SARS-CoV-2 characterized in this study.

## Discussion

In this study, we conducted a cross-sectional survey in dogs and cats in private animal hospitals from June–September 2021. During investigation period, we identified SARS-CoV-2 delta variant of concern (B.1.617.2) in dogs and cats with the occurrence of 20.0% (1/5) in dogs and 7.1% (1/14) in cats (16.7%; 2 from 12 COVID-19 households). Notably, the occurrence of SARS-CoV-2 may relate to timing of sample collection, health condition of animals and close contact with the owners^1,22,23^. The B.1.617.2 lineage was a predominant lineage of the recent 4th wave of COVID-19 outbreaks in Thailand^13^. Comparing to the previous study in Thailand, SARS-CoV-2 infection in dogs and cats in April to May 2021, dogs and cats were infected with the prevalence of 8.6% (3/35) in dogs and 11.1% (1/9) in cats (23.5%; 4 from 17 COVID-19 households) and SARS-CoV-2 of the Alpha variant (B.1.1.7 lineage) was identified^1^. Notably, SARS-CoV-2 delta variant (B.1.617.2) infection in dogs was first reported in Kansas, USA and Barcelona, Spain in mid-2021^17,18^. In cats, SARS-CoV-2 delta variant (B.1.617.2) infection in cats was reported in Harbin, China in September 2021^19^. Moreover, SARS-CoV-2 delta variant infection have been reported in Asiatic lions in zoological park in India and white-tail deer in Canada^24,25^.

Virological test for SARS-CoV-2 showed that viral RNA could be detected from nasal and oral swabs of a cat and a dog at day 1 (4–5 days after the owner reported COVID-19 positive). It is interesting to note that SARS-CoV-2 RNA could also be detected in animal hair of cat at day 3 and environmental samples (hair, water container and floor) of dog at day 3 and day 7. Similarly, in previous reports, SARS-CoV-2 RNA could be found in environmental samples e.g. water container and cage floor of domestic pet as well as dust and air samples in mink farms^1,26^. Serological test for SARS-CoV-2 antibodies in this study showed that a cat developed SARS-CoV-2 antibodies since day 14 (by ELISA, sVNT and pVNT) after SARS-CoV-2 RNA detection. While a dog was positive for SARS-CoV-2 antibodies at day 9 (by pVNT) and day 24 (by sVNT and pVNT). Our findings in agreement with previous studies that dogs and cats can develop antibodies against SARS-CoV-2 as early as 7 to 14 days post infection^5,27,28^. For example, our previous studies showed that SARS-CoV-2 antibodies could be detected from dogs at day 11–23 and from cats at day 6 after SARS-CoV-2 RNA detection. Notably, control dog and cat sera and pre-COVID-19 sera have been tested in our previous report^1^. However, the timing for serum sample collection when the animal might have been exposed to COVID-19 owners could influence the antibodies titer and the discrepancy between the result of ELISA, sVNT and pVNT.

Phylogenetic analysis showed that the cat and dog SARS-CoV-2 were grouped into delta variant of concern (B.1.617.2) sublineage AY.30 (B.1.617.2.30) and AY.85 (B.1.617.2.85), respectively. Both sub-lineages are the predominant sub-lineages of delta variant in the 4th wave of COVID-19 outbreaks in Thailand with the prevalence of 71.0% and 77.0%, respectively (Pangolin lineage; https://cov-lineages.org/lineage_list.html). Unlike previous studies, SARS-CoV-2 delta variant, sub-lineage AY.3 was responsible for COVID-19 in dog in the US and sub-lineage AY.43 in dog in Spain^17,18^. Genetic mutation analysis of the cat and dog SARS-CoV-2 showed that all mutations agreed with those of delta variant delta variant of concern (B.1.617.2) including the mutations at the N-terminal-domain (NTD), the receptor binding motif (RBM), the subdomain 2, S1 unit and heptad repeat 1. Since SARS-CoV-2, B.1.617.2 lineage has higher transmissibility rates than the B1.1.7 viruses, thus, mutations on spike protein in relation to transmissibility or host adaptation of animal SARS-CoV-2 needed further investigation^29^. A previous study reported role of mutations on spike gene may not in relation to host-adapted mutations^30^. Notably, unlike previous study, M1227L on S gene was not observed in our SARS-CoV-2 from dog. It speculated that this point mutation may relate to S protein destabilization and transmission from human-to-dog^17,31^.

In conclusion, results from phylogenetic and mutation analysis suggested that the virus infecting dog and cat in this study originated from a local outbreak cluster of delta variant AY.30 and AY.85. Notably, AY.30 and AY.85 sub-lineages are predominately found in Thailand. In Thailand, most veterinary clinics and hospitals have followed COVID-19 management guideline provided by the department of livestock development, Thailand. Since most SARS-CoV-2 infected animals are asymptomatic or less symptomatic. History of close contact between owners and domestic animals in COVID-19 positive household is crucial for veterinary practitioners to monitor SARS-CoV-2 infection in domestic animals. This study is the first to report SARS-CoV-2 delta variant infection in domestic dogs and cats in Thailand. Our finding supports routine surveillance of SARS-CoV-2 in domestic animals and raises more awareness on frequent spill-over of variant of concerns due to high human-animal interface.

## Material and methods

### Sample collection from domestic dogs and cats for SARS-CoV-2

During June–September 2021, we conducted SARS-CoV-2 survey in domestic dogs and cats living in Bangkok and the vicinities. Samples from dogs and cats were collected from participating veterinary clinics and hospitals. In total, we collected 225 samples from dogs (n = 105) and cats (n = 120) from COVID-19 positive household (n = 12) and unknown status households (n = 187) (Tables 1 and 2). We collected nasal swabs (dog; n = 8; cat; n = 16), oral swabs (dog; n = 102, cat; n = 117) and rectal swabs (dog; n = 104, cat; n = 120) from the animals by using flocked nylon swab (Copan®, California, USA). The swab samples were placed in RNA protect® (Qiagen LLC, Maryland, USA) and transported to the laboratory within 24 h. In this study, we followed up SARS-CoV-2 positive dog (n = 1) and cat (n = 1). During follow up, we collected nasal swabs (n = 9), oral swabs (n = 9), rectal swabs (n = 9) from the animals. In addition, environmental swabs, hair (n = 7), water container (n = 7), and floor (n = 7) were also collected (Table 3). We collected blood sample (n = 6 serum; 2 time points from a cat and 4 timepoints from a dog) from SARS-CoV-2 positive animals (Table 4). This study was conducted under the approval of the Institutional Animal Care and Use Committee (IACUC) of the Faculty of Veterinary Science, Chulalongkorn University, Thailand (approval No. 2031035). All methods were carried out in accordance with relevant guidelines and regulations.

### Detection of SARS-COV-2 RNA

Viral RNA extraction was performed by using GENTi—Automated Nucleic Acid Extraction System (GeneAll® Seoul, Korea). For the detection of SARS-CoV-2 RNA, realtime RT-PCR assays based on specific primers and probes (N2, E and RdRp) were performed following CDC and WHO recommendations^20,21^. In brief, a total 25 µl reaction contained 2 µl of RNA, 12.5 µl of 2X reaction buffer of the SuperScript® III Platinum® One-Step Quantitative RT-PCR System (Invitrogen, California, USA), 1 µl of reverse transcriptase/Platinum Taq, 0.8 mM MgSO4, 0.8 µM each primer and probe and RNase-free water. Realtime RT-PCR reaction was setup at 50 °C for 15 min, followed by 95 °C for 2 min and 45 cycles of 95 °C for 15 s, and 58 °C for 30 s (E and RdRP genes) or 60 °C for 30 s (N2 gene). Samples with a Ct value of < 40 were considered positive. Due to the limitation of institute and IACUC approval, virus isolation did not perform in the study.

### Characterization of SARS-CoV-2

The RNA samples from the nasal swab of a cat (C27516) with SARS-CoV-2 positive collected on 15 July 2021 (Ct value 20.66) and the nasal swab of a dog (CU27791) collected on 12 September 2021 (Ct value 19.06) were subjected to whole genome ssequencing by using Oxford Nanopore. We used the ARTICS nCoV-2019 sequencing protocol V3 (LoCost) to amplify viral genome. In brief, diluted RNA (8 µl) was mixed with 2 µl of LunaScript® RT SuperMix (NEB, Ipswich, MA, USA). The cDNA synthesis was performed at 25 °C for 2 min, 55 °C for 10 min and 95 °C for 1 min. The ARTIC protocol using two pools of the SARS-CoV-2 primers for multiplex PCRs was performed by using Q5® Hot Start High-Fidelity DNA polymerase (NEB, MA, USA) with PCR reaction at 98 °C for 30 s and 35 cycles of 98 °C for 15 s and 65 °C for 5 min. After PCR amplification, library preparation was performed following the Oxford Nanopore rapid sequencing kit (SQK-RAD004) with ARTIC SARS-CoV-2 genome sequencing protocol^32,33^. In brief, 7.5 µl of pooled PCR products (10 µl of pool 1 and 10 µl of pool 2) was added to 2.5 µl of fragmentation mix. Then the mixture was incubated at 30 °C for 1 min, 80 °C for 1 min, and 4 °C for 30 s. The mixture was cleaned by AMPure XP Bead Cleanup (Beckman Coulter, CA, USA) and eluted with 15 µl of 10 mM Tris–HCl pH 8.0. The mixture was loaded into Oxford Nanopore MinION device under MinKNOW version 19.12.5 software (Oxford nanopore technologies, Oxford, UK) (https://nanoporetech.com/nanopore-sequencing-data-analysis)^34^. After sequencing, nucleotide sequences were filtered using the sequencing summary file under the following parameters: minimum read length ≥ 500 nt and read quality ≥ 7. The qualify reads were conversed from “Fast5” into “Fastq” format by using the GPU version of the Nanopore Guppy basecaller (v3.4.4) tool. The Fastq format sequences were assembled using the genome detective program^35^ and de-novo approach with Qiagen CLC Genomics Benchwork version 20.0.4 software (QIAGEN, CA, USA) (https://digitalinsights.qiagen.com/ products/qiagen-clc-main-workbench/). Whole genome sequences of the SARS-CoV-2 were deposited into the GenBank (OK555092 and OK539641) and GISAID (EPI_ISL_5320246 and 5315539).

### Phylogenetic analysis of SARS-CoV-2

Whole genome sequences of SARS-CoV-2 were subjected to lineage classification by using the COVID-19 sequences of the Phylogenetic Assignment of Named Global Outbreak Lineages (PANGOLIN) (https://cov-lineages.org/resources/pangolin.html). Phylogenetic analysis of SARS-CoV-2 was performed by comparing nucleotide sequences of 289 SARS-CoV-2 genomes available in the GISAID and GenBank database. The 5′ and 3′ untranslated regions were trimmed with at least 95% reference genome coverage (Wuhan-Hu-1) (at least 29,000 bp in length). The dataset alignment was performed by using the MAFFT FFT-NS-2 algorithm with default parameter settings^36^. The maximum likelihood tree was constructed by using IQ-TREE version 2.1.3 (http://www.iqtree.org/)^37^ using the GTR + Γ model of nucleotide substitution^38^, default heuristic search options, and ultrafast bootstrapping with 1000 replicates^39^. Tree was visualized by iTOL version 6.0 (https://itol.embl.de/)^40^. Lineage classification was performed by using the Pangolin tool^41^. Genetic mutation analysis of the SARS-CoV-2 was performed by comparing deduced amino acids of each gene of the viruses based on variant classifications and definitions^42,43^.

### Detection of SARS-CoV-2 antibodies

The ID Screen® SARS-CoV-2 Double Antigen Multi-species ELISA kit (ID VET, Montpellier, France) was used to detect IgG antibodies against N protein of the SARS-CoV-2 virus in animal sera. We performed the ELISA test according to the manufacture’s recommendation. In brief, 25 µl of each serum sample was diluted at 1:1 ratio with the dilution buffer and added to each well. The 96-well plate was incubated at 37 °C for 45 min. The plate was washed with 300 µl of washing solution and 100 µl of N protein recombinant antigen horseradish peroxidase (HRP) conjugate was added to each well and incubated at 25 °C for 30 min. The plate was washed 3 times with 300 µl of washing solution. After washing, 100 µl of the substrate solution was added into each well and incubated at 25 °C for 20 min. Then, 100 µl of the stop solution was added. The reaction was read and recorded for the optical densities (O.D.) at 450 nm. The O.D. was calculated as the S/P percentage (S/P%). If S/P% value greater than or equal to 60% is considered positive, while sample with S/P% between 50 and 60% is considered as doubtful, and sample with S/P% < 50 is negative.

The cPass SARS-CoV-2 Neutralization Antibody Detection Kit (GenScript Biotech, Jiangsu, China) was used to detect neutralizing antibodies. The assay detects SARS-CoV-2 antibodies by measurement of antibody-mediated inhibition of SARS-CoV-2 RBD-ACE2 interaction. In brief, 50 µl of diluted 1:10 serum was incubated with 50 µl of HRP-conjugated RBD and incubated at 37 °C for 30 min. The 100 µl of treated serum then added to ACE2-coated ELISA plate and incubated at 37 °C for 15 min. Then the uncaptured substrate was washed out by using 260 µL of washing solution for four times. Colorimetric signal was developed by using TMB substrate at 25 °C for 15 min. Absorbance reading at 450 nm was acquired using a microplate reader immediately after adding stop solution. The percentage inhibition was calculated. The sample with % inhibition ≥ 20% indicate the presence of SARs-CoV-2 neutralizing antibody, otherwise are negative^44^.

Pseudotype virus neutralization test was performed by using a SARS-CoV-2 lentiviral pseudotype and HEK293T expressing human ACE2. The hACE2 expressing target cells was produced by stable transduction of HEK293T cell with lentiviral vector harbouring hACE2 gene (pHAGE-EF1alphaInt-ACE2-WT, BEI Resources, VA, USA; NR-52512) and enriched by magnetic cell sorting using mouse anti-hACE2 (Sino-biological, China) and goat anti-mouse IgG microbeads with MACS LS column (Miltenyi Biotec Asia Pacific, Singapore ). The SAS-CoV-2 lentiviral pseudotype was produced by co-transfecting plasmids pSPAX2 (Addgene, MA, USA; plasmid # 12,260), pCCGW and pHDM-SARS-CoV-2 spike (BEI Resources, VA, USA; NR-53742) into HEK293T cell. The pHDM-SARS-CoV-2 spike vector encodes the codon optimized spike gene of SARS-CoV-2 strain Wuhan-Hu-1 (GenBank NC_045512). All serum samples were heat-inactivated in a biosafety cabinet at 56 °C for 60 min and twofold serially-diluted covering 1:20 to 1:40,960 in DMEM supplemented with 10% fetal bovine serum. The sera were incubated with 50 µL of 100 TCID_50_ of the SARS-CoV-2-lentiviral pseudotype at 37 °C for 1 h. Then, 50 µL of 1 × 10^4^ HEK293T-hACE2 cell was added into the mixture and incubated at 37 °C for 48 h. A dilution at which the 50% of infection as compared to anti-SARS-CoV-2-negative serum is inhibited (IC50) was used as test cut-off. The plant-based anti-SARS-CoV-2 antibodies; H4 was used as antibody positive control^45,46^.

### Ethics statement

The Institutional Animal Care and Use Committee of the Faculty of Veterinary Science, Chulalongkorn University, Thailand approved animal study (IACUC No. 2031035). All methods were carried out in accordance with relevant guidelines and regulations. The study complies with the ARRIVE guidelines. The IACUC committee of the Faculty of Veterinary Science, Chulalongkorn University, Thailand approved the informed consent. The informed verbal consent was obtained from all pet owners and private animal hospital staff after explaining the objectives and benefits of the study during sample collection.

## Supplementary Information

Supplementary Information.

## Data Availability

The authors declare that the data supporting the findings of this study are available within the article and its technical appendix files. The nucleotide sequence data have been deposited at the GenBank with accession numbers OK555092 and OK539641 and GISAID with accession numbers EPI_ISL_5320246 and EPI_ISL_5315539.
