# Supplementary material for: SARS-CoV-2 delta variant infection in domestic dogs and cats, Thailand
Source: Sci Rep. 2022 May 19;12:8403. doi: 10.1038/s41598-022-12468-y (PMC9117851; doi:10.1038/s41598-022-12468-y)
Supplement: Supplementary file 1 — Supplementary Information. [file 41598_2022_12468_MOESM1_ESM.docx]

**Supplement Material**

# SARS-CoV-2 delta variant infection in domestic dogs and cats, Thailand

Waleemas Jairak^1,2,3^, Ekkapat Chamsai^1,2^, Kitikhun Udom^1,2^, Kamonpan Charoenkul^1,2^

Supassama Chaiyawong^1,2^, Navapon Techakriengkrai ^1,4^, Ratanaporn Tangwangvivat ^5^,

Kamol Suwannakarn^6^, Alongkorn Amonsin^1,2^*

**Author affiliations:**

^1^ Center of Excellence for Emerging and Re-emerging Infectious Diseases in Animals, and One Health Cluster, Faculty of Veterinary Science, Chulalongkorn University, Bangkok, Thailand
^2^ Department of Veterinary Public Health, Faculty of Veterinary Science, Chulalongkorn
University, Bangkok, Thailand
^3^ Bureau of Research and Conservation, Zoological Park Organization, Bangkok, Thailand

^4^ Department of Veterinary Microbiology, Faculty of Veterinary Science, Chulalongkorn University, Bangkok, Thailand

^5^ Coordinating Unit for One Health, Division of Communicable Diseases, Department of Disease Control, Nonthaburi, Thailand

^6^ Department of Microbiology, Faculty of Medicine Siriraj Hospital, Mahidol University, Bangkok, Thailand

**Supplement Table 1.** Result of Nanopore sequencing of SARS-CoV-2 from dog and cat, Thailand

| Animal | Strain | GenBank accession No. | GISAID  accession No. | # mapped reads | % mapped reads* | Average length (bp) | Total  nt sequence | Genome size (bp) | Coverage (x) |
| --- | --- | --- | --- | --- | --- | --- | --- | --- | --- |
| Cat | CU 27516 | OK555092 | EPI_ISL_5320246 | 115,533 | 66.56 | 338.33 | 39,088,741 | 29,861 | 1,309 |
| Dog | CU 27791 | OK539641 | EPI_ISL_5315539 | 191,132 | 85.70 | 356.97 | 68,229,056 | 29,704 | 2,296 |

*Whole-genome alignment tools; CLC Genomics Workbench version 20.0.4 (Qiagen A/S, Vedbæk, Denmark)

**Supplement Table 2.** BLAST analysis of genome sequences of SARS-CoV-2 from cat and dog with reference viruses

| Virus | GISAID/GenBank# | Location | Species | Year | Lineage | Nucleotide similarities (%) | | | | | | | | | | | | |
| --- | --- | --- | --- | --- | --- | --- | --- | --- | --- | --- | --- | --- | --- | --- | --- | --- | --- | --- |
|  |  |  |  |  |  |  | | | | | | | | | | | | |
|  |  |  |  |  |  | WGS | ORF1ab | ORF1a | S | ORF3a | E | M | ORF6 | ORF7a | ORF7b | ORF8 | N | ORF10 |
|  |  |  |  |  |  |  |  |  |  |  |  |  |  |  |  |  |  |  |
| CU27516 ^a^ | EPI_ISL_5320246 | Thailand | Cat | Jul-21 | B.1.617.2 |  |  |  |  |  |  |  |  |  |  |  |  |  |
| Wuhan-Hu-1 | NC_045512.2 | China | Human | Dec-19 | B | 99.88% | 99.93% | 99.92% | 99.79% | 99.88% | 100% | 99.85% | 100% | 99.17% | 100% | 100% | 99.60% | 100% |
| QEUH-B80FF1 | EPI_ISL_661814 | UK | Human | Nov-20 | B.1.1.7 | 99.81% | 99.90% | 99.89% | 99.68% | 99.88% | 100% | 99.85% | 100% | 99.17% | 100% | 99.16% | 99.12% | 100% |
| NHLS-UCTGS  -7500-KRISP | EPI_ISL_696476 | S. Africa | Human | Nov-20 | B.1.351 | 99.81% | 99.90% | 99.88% | 99.59% | 99.64% | 99.56% | 99.70% | 100% | 99.17% | 100% | 100% | 99.52% | 100% |
| TN-CL-423-S234-R1-001 | EPI_ISL_2463770 | India | Human | May-21 | B.1.617.2 | 99.92% | 99.92% | 99.93% | 99.95% | 99.88% | 100% | 100% | 100% | 99.73% | 100% | 99.72% | 99.84% | 100% |
| AFRIMS-COV2513-2021 | MZ888556.1 | Thailand | Human | Aug-21 | B.1.617.2 | 99.98% | 99.99% | 99.98% | 99.95% | 100% | 100% | 100% | 100% | 100% | 100% | 100% | 99.92% | 100% |
| AFRIMS-COV3783-2021 | OK626714.1 | Thailand | Human | Oct-21 | B.1.617.2 | 99.88% | 99.88% | 99.86% | 99.92% | 99.88% | 99.56% | 99.85% | 100% | 99.73% | 99.23% | 100% | 99.76% | 100% |
| NHSAD-21-0019 | EPI_ISL_2821077 | India | Lion | May-21 | B.1.617.2 | 99.92% | 99.93% | 99.93% | 99.95% | 99.88% | 100% | 100% | 100% | 100% | 100% | 100% | 99.84% | 100% |
| NHSAD-21-0011 | EPI_ISL_2821078 | India | Lion | May-21 | B.1.617.2 | 99.92% | 99.93% | 99.93% | 99.95% | 99.88% | 100% | 100% | 100% | 100% | 100% | 100% | 99.84% | 100% |
| CU27791^b^ | EPI_ISL_5315539 | Thailand | Dog | Sep-21 | B.1.617.2 | 99.87% | 99.89% | 99.86% | 99.92% | 99.64% | 99.56% | 99.85% | 100% | 99.73% | 99.23% | 100% | 99.76% | 100% |
|  |  |  |  |  |  |  |  |  |  |  |  |  |  |  |  |  |  |  |
|  |  |  |  |  |  | WGS | ORF1ab | ORF1a | S | ORF3a | E | M | ORF6 | ORF7a | ORF7b | ORF8 | N | ORF10 |
|  |  |  |  |  |  |  |  |  |  |  |  |  |  |  |  |  |  |  |
| CU27791^b^ | EPI_ISL_5315539 | Thailand | Dog | Sep-21 | B.1.617.2 |  |  |  |  |  |  |  |  |  |  |  |  |  |
| Wuhan-Hu-1 | NC_045512.2 | China | Human | Dec-19 | B | 99.86% | 99.92% | 99.92% | 99.82% | 99.52% | 99.56% | 99.70% | 100% | 99.45% | 99.23% | 100% | 99.68% | 100% |
| QEUH-B80FF1 | EPI_ISL_661814 | UK | Human | Nov-20 | B.1.1.7 | 99.80% | 99.89% | 99.89% | 99.71% | 99.52% | 99.56% | 99.70% | 100% | 99.45% | 99.23% | 99.16% | 99.20% | 100% |
| NHLS-UCTGS  -7500-KRISP | EPI_ISL_696476 | S. Africa | Human | Nov-20 | B.1.351 | 99.80% | 99.89% | 99.87% | 99.61% | 99.27% | 99.12% | 99.55% | 100% | 99.45% | 99.23% | 100% | 99.60% | 100% |
| TN-CL-423-S234-R1-001 | EPI_ISL_2463770 | India | Human | May-21 | B.1.617.2 | 99.88% | 99.89% | 99.88% | 99.92% | 99.51% | 99.56% | 99.85% | 100% | 100% | 99.23% | 99.72% | 99.92% | 100% |
| AFRIMS-COV2513-2021 | MZ888556.1 | Thailand | Human | Aug-21 | B.1.617.2 | 99.86% | 99.88% | 99.85% | 99.92% | 99.64% | 99.56% | 99.70% | 100% | 99.73% | 99.23% | 100% | 99.68% | 100% |
| AFRIMS-COV3783-2021 | OK626714.1 | Thailand | Human | Oct-21 | B.1.617.2 | 99.98% | 99.98% | 99.97% | 100% | 99.76% | 100% | 100% | 100% | 100% | 100% | 100% | 100% | 100% |
| NHSAD-21-0019 | EPI_ISL_2821077 | India | Lion | May-21 | B.1.617.2 | 99.88% | 99.89% | 99.88% | 99.92% | 99.51% | 99.56% | 99.85% | 100% | 100% | 98.99% | 100% | 99.92% | 100% |
| NHSAD-21-0011 | EPI_ISL_2821078 | India | Lion | May-21 | B.1.617.2 | 99.88% | 99.89% | 99.88% | 99.92% | 99.51% | 99.56% | 99.85% | 100% | 100% | 98.99% | 100% | 99.92% | 100% |
| CU27516 ^a^ | EPI_ISL_5320246 | Thailand | Cat | Jul-21 | B.1.617.2 | 99.87% | 99.89% | 99.86% | 99.92% | 99.64% | 99.56% | 99.85% | 100% | 99.73% | 99.23% | 100% | 99.76% | 100% |
|  |  |  |  |  |  |  |  |  |  |  |  |  |  |  |  |  |  |  |
